# Translation and Validation of the Malay Version of the Parents’ Satisfaction Scale (PSS-M) for Assessment of Caregivers’ Satisfaction with Health Care Services for Children with Autism Spectrum Disorder

**DOI:** 10.3390/ijerph15112455

**Published:** 2018-11-04

**Authors:** Nik Aida Nik Adib, Mohd Ismail Ibrahim, Azriani Ab Rahman, Raishan Shafini Bakar, Nor Azni Yahaya, Suria Hussin, Wan Nor Arifin

**Affiliations:** 1 Department of Community Medicine, School of Medical Sciences, Universiti Sains Malaysia, Kubang Kerian, Kota Bharu, 16150 Kelantan, Malaysia; eyedasyukran@gmail.com (N.A.N.A.); azriani@usm.my (A.A.R.); 2 Department of Psychiatric, School of Medical Sciences, Hospital Universiti Sains Malaysia, Kubang Kerian, Kota Bharu, 16150 Kelantan, Malaysia; raishanshafini@usm.my; 3 Department of Pediatric, Hospital Raja Perempuan Zainab II, Kota Bharu, 15150 Kelantan, Malaysia; drazni@yahoo.co.uk; 4 Department of Psychiatric, Hospital Raja Perempuan Zainab II, Kota Bharu, 15150 Kelantan, Malaysia; suriahussin74@yahoo.com.my; 5 Unit of Biostatistics and Research Methodology, School of Medical Sciences, Universiti Sains Malaysia, Kubang Kerian, Kota Bharu, 16150 Kelantan, Malaysia; wnarifin@usm.my

**Keywords:** PSS-M, caregivers’ satisfaction, confirmatory factor analysis, validity

## Abstract

Background: A Malay version of Parent Satisfaction Scale (PSS-M) is needed to investigate the factors contributing to the Malay caregivers’ satisfaction with health care management for children with autism spectrum disorder (ASD). The aim of the study is to translate and validate the questionnaire to assess the caregivers’ satisfaction on health care services. Methods: A cross-sectional study was conducted among 110 caregivers of children with ASD aged between 2 and 17 years old that received treatment at two tertiary care centres in Kelantan. Permission to use the original version of the PSS questionnaire was obtained. The original English version of the PSS was translated into a Malay version following the 10 steps proposed by an established guideline. Pre-testing of the PSS was carried out with 30 caregivers before confirmatory factor analysis (CFA) was established using 110 caregivers. They were asked to assess their understanding of the questionnaire. The one-dimensional questionnaire consists of 11 items, including staff attitudes, availability of staff, supportiveness, and helpfulness. The 5-point Likert scale provided ratings from 1 (strongly disagree) to 5 (strongly agree). Confirmatory factor analysis was performed using a robust maximum likelihood estimator. Results: The analysis showed model fit data with good reliability. Conclusion: The PSS-M shows overall model fitness based on specific indices, with good construct validity and excellent absolute reliability to determine the satisfaction level of caregivers of children with ASD with respect to health care services.

## 1. Introduction

Measurement of the satisfaction level of caregivers of children with autism spectrum disorder (ASD) in health care is essential to give evidence that the provided services met caregivers’ expectations of desirable care [1]. It is essential for determining the accessibility and equality of healthcare delivery widely accepted and sustained in various healthcare settings. Client satisfaction can be based on numerous factors, such as healthcare settings, technical management, and features of interpersonal care and staff interactions [2]. Among these factors, most authors believe that the patient–staff interaction has the best effect on client satisfaction [3,4,5,6].

ASD is a neurodevelopmental disorder characterized by deficits in social communication, social interaction, and restricted, repetitive patterns of behaviours, interests, and activities beginning in childhood. It often associated with co-morbidities: intellectual impairment, disruptive behaviours, attention difficulties, aggression, poor eating, sleep problems, epilepsy, gastrointestinal problems, and motor coordination issues [7,8]. The term “spectrum” in this case refers to symptoms that can range from mild to severe and will affect the children differently even though they may have similar symptoms depending on the severity [8].

**Figure 1:**
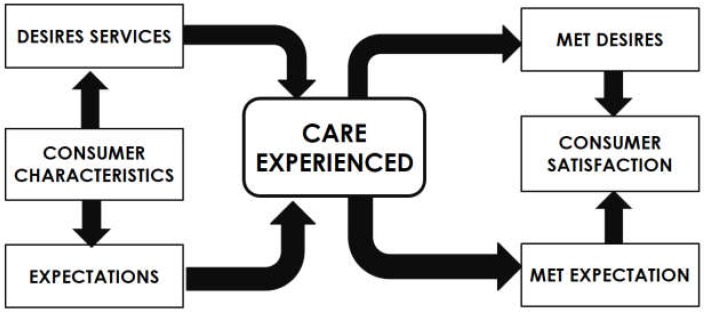
Consumer satisfaction model, modified from Gerkensmeyer et al. [10,11].

The original author developed the consumer satisfaction model prior to this study to better define parent satisfaction, and to examine the underlying determinants of parent satisfaction [9]. In the model (Figure 1) the care experience is measured by the services parents or caregivers received. Met desires and met expectations, for instance, are an incorporated element to measure the care experienced. The consumer is satisfied with the services provided when the elements of met desired and met expectations are fulfilled. The consumer satisfaction model includes consumers’ perceptions about what is expected or valued as a baseline with which to compare their perceptions of services received. Satisfaction was determined by the difference between perceived actual services and consumers’ perceptions of ideal, expected, or deserved services [9]. The consumers’ characteristics as illustrated in Figure 1 are proposed to influence how consumers define their situation of having a child with general mental health problems. Consumer characteristics and a definition of the situation are proposed to influence consumers’ desired services, perceived care needs, and expectations on services. Desired services, perceived care needs, and expectations serve as consumers’ psychological standards for a comparison with the care they experience. Comparing consumers’ perceived expectations, needs, or desires with perceptions of care experienced is proposed to result in consumers’ judgments about their level of met desires, met needs, and met expectations. Met desires, met needs, and met expectations are proposed to influence satisfaction. The Parent Satisfaction Scale (PSS) questionnaire consequently only measured the construct of parent satisfaction. Met desires, met needs, and met expectations are predicted to be significantly related in the PSS [10,11].

Knowledge of caregivers’ perceptions and satisfaction with the services is vital to improve health care services, as caregivers have substantial role in their children’s overall development. Caregivers of children with ASD reported that they were overwhelmed by the impact of health care procedures and management on their children. The majority of caregivers reported that service provision remained far from their expectations, and claimed that the management provided by the health care system was a significant source of stress [12,13,14].

Data from Asian countries are extremely limited and very minimal. As for now, research on the caregivers’ stress and satisfaction on the health care management of children with ASD is mostly confined to those living in Canada, the United States, and Europe [14,15,16,17].

Of the 79 publications related to ASD in Malaysia, there are no studies assessing caregivers’ satisfaction on the health care management of ASD. Only 12.0% are related to caregiver psychology. Most interventions assess only in terms of child outcome, thus ignoring caregiver factors, which may have an influence on effect on the intervention given [18].

According to the latest National Health System Review (NHSR), a study on client satisfaction is highly recommended and essential in order to improve the efficiency of the current services [19]. Therefore, input from primary caregivers about their perception toward health care management is an important aspect of evaluating ASD services.

In addition, knowing the elements that might increase and decrease caregivers’ satisfaction might improve the adherence of caregivers to the clinical management of ASD, improving the quality of life of all of them.

There are many tools have been developed worldwide to measure the satisfaction level related to health care services. However, the PSS is the only instrument out of 37 identified instrument that has been appropriately validated. The validation was conducted by a group of researchers from the Indiana University School of Nursing, USA [20]. Thus, the PSS questionnaire was chosen as a tool to assess the satisfaction level of the client among Malaysian caregivers. There is a need to translate the PSS, since most of the respondents involved in the current study are Malay. It is important to suit with linguistic appropriateness so that the respondents will give a valid and reliable result [21,22].

Having a valid Malay version questionnaire will help the respondents to give their actual experiences and opinions. This will assist in significantly changing the entire health care system, especially the management of children with ASD. In addition to that, it might help the health care provider to have a greater understanding of caregivers’ needs in order to improve their treatment adherence. Its importance in determining the accessibility and equality of health care delivery has been widely accepted and sustained in various health care settings.

The Parent Satisfaction Scale (PSS) was a scientific tool designed by Gerkensmeyer and Austin to measure parents’ satisfaction with child’s mental health services, primarily focusing on staff’s interpersonal interactions [10]. The PSS is a one-dimensional questionnaire that consists of several items assessing staff attitudes, availability, supportiveness, and helpfulness (for instance inclusion of parents and informing parents) [10]. It showed an excellent internal consistency (Cronbach’s alpha = 0.96), significant construct validity (i.e., parent satisfaction according to meet desires, needs, and expectations), and convergent validity with the Client Satisfaction Instrument (r = 0.86, *p* < 0.001). The PSS used in paediatric therapies services because of its rigorous development, excellent psychometric properties, and a clear conceptual framework of parent satisfaction that agrees with the unique and varied aspects of these services.

This study aims to translate the PSS into Malay version and validate the Malay version among caregivers who have children with ASD in Kelantan.

## 2. Materials and Methods

### 2.1. Study Setting and Participants

A cross-sectional study was conducted among 110 caregivers for two months starting from February 2018 among caregivers of children with ASD who received treatment from all tertiary centres in Kelantan, namely Hospital Raja Perempuan Zainab II (HRPZ II) and Hospital Universiti Sains Malaysia (HUSM). According to [23], the minimum sample size required for this study was 100 samples. In view of original PSS consist of one construct and 11 items on average, the all-item communality was more than 0.6. After taking into consideration a 10.0% drop-out rate, the sample size required for this study was 110 samples [23].

The duration of study includes data collection and data analysis. Data collection required a more extended period to get their consent and need to suit their appointment time at the study centre either monthly or even longer depending on the spectrum and complexity of the autism. A list of confirmed children with ASD which fulfilled the inclusion and exclusion criteria (with complete contact numbers for caregivers) from both centres was obtained. Simple random sampling method was used to obtain the appropriate number of respondents according to the calculated sample size. Those selected were contacted and asked to join the research project. The consent form was given to those who agreed to participate in the study during appointment date of their child’s therapy. Caregivers that lived with and had taken care of children with ASD since birth and who had children with ASD aged between two and 18 years old were included in this study. Those main caregivers whom were illiterate and/or known to have an underlying psychiatric problem and/or received therapies at private centres were excluded from the study. In addition to that, the children for whom the diagnosis was not yet confirmed during the study period were also excluded. The consenting caregivers were given PSS questionnaires that had been translated into the Malay version and that could be completed within 15 min.

The main caregiver was defined as the person who was primarily responsible for development of the child and was involved most in the management either during hospital visit, consultation with specialist, and follow up for all kinds of clinical interventions. They could be biological parents or individuals who had taken care of the child since birth.

### 2.2. Parent Satisfaction Scale (PSS)

The one-dimensional PSS is a self-administered questionnaire consisting of 11 statements or items. Each statement had five Likert scale responses ranging from one to four with score 1 representing strongly disagree and score 5 representing strongly agree. Higher scores indicate higher level of satisfaction on the interpersonal relationship with professional. The respondents were asked to choose one best response for each statement.

The PSS has been translated into other languages in the past. A validation study conducted in Tagalog language among 125 caregivers of patients receiving therapies in a clinic in Manila, Philippines also showed excellent internal consistency similar to that of the original version in terms of the raw ordinal coefficient value (α = 0.96) and each item-total correlation (α = 0.79 to 0.89), with good absolute reliability (standard error of the mean (SEM) = 0.11), and moderate to substantial agreement with k values (ranging from 0.56 to 0.72) [24].

The process of translation followed the guideline proposed by [22]. This guideline comprised of 10 stages, including:

(1) Preparation
(2) Forward Translation
(3) Reconciliation
(4) Back Translation
(5) Back Translation Review
(6) Harmonisation
(7) Cognitive Debriefing
(8) Review of Cognitive Debriefing Results and Finalisation
(9) Proof-Reading
(10) Final Report

The translation of PSS was conducted according to this stage as shown below:

(1) Preparation

Preparation included the first works that were carried out before the translation work begin. At this stage, the translators of PSS were identified, contacted, and informed regarding the purpose of this research. Researchers and translators also determined deadlines for each stage during this stage

(2) Forward Translation

The forward translation involved translation of PSS from the English language into the target Malay language questionnaires from the English language into the target language (Malay). This process was performed by two independent translators designated as Translator One and Translator Two. The first identified translator was a psychiatrist from the HRPZ II (Translator One). A teacher from the linguistics department of the Universiti Sains Malaysia (USM) was identified as Translator Two. Both translators were fluent in both English and Malay.

Both translators were asked to note any items, sentences or statement in the questionnaire which were difficult or impossible to translate. Both of translators were conducted their translations independently. In addition to the original questionnaire in hard copy, a soft copy of PSS questionnaire in the format of Microsoft Word was also provided to facilitate the translation process.

(3) Reconciliation

The purpose of reconciliation was to compare and merge both forward translations into a single forward translation. The committee reviewed both forward translations. The review committee consisted of a public health lecturer, a psychiatrist, and a statistician who were fluent in both the English and Malay languages.

Both versions of the forward translations were compared with the original PSS. The reconciliation process was completed by comparing each sentence in both translations. Words or phrases that were not relevant within the Malaysian context were replaced with alternative words or phrases. The content validity of the questionnaire was also determined at this stage. 

At the end of the meeting, one common forward translation of the PSS was produced and agreed upon by the review committee to be used in the following stage.

(4) Back Translation

Back translation is the translation of the intended new language version back into original language. The common forward translation of the questionnaire was sent to another two independent translators to be translated back into the English language. The identified as Translator One was a child psychologist from the USM. Translator Two was a lecturer from Women’s Health and Development Unit, USM. Both translators were blinded to the original questionnaire and the purpose of the study. Both translators were fluent in both languages. The period taken by both translators for back translation was about two weeks.

(5) Back Translation Review

Back translation review was performed to compare the back-translated version of the questionnaire with the original to highlight and investigate discrepancies between the original and the reconciled translation. At this stage, the back translation was examined against the original English version by the same review committee that synthesized the forward translation.

Three types of equivalences were established: conceptual equivalence, item equivalence, and semantic equivalence. The conceptual equivalence was the degree to which a given concept was present in both source and targeted culture [25]. The conceptual equivalence was established based on the consensus of expert opinion from a member of the committee after considering the critical element of caregiver satisfaction during the health care management of children with ASD. Item equivalence is concerned with whether the specific items are relevant and acceptable in the target populations [26]. Semantic equivalence of PSS between the original English version and translated version in Malay was assured through the process of forward and backward translation.

In general, the review committee was satisfied with the equivalences between the common forward translation of PSS and the original English version of PSS. The common forward translation hence was regarded as the pre-final PSS-M to be tested in the next stage.

(6) Harmonisation

Harmonization is the stage where a comparison is made between the back translation of Malay language versions and with the original instrument to highlight the discrepancies in between the original and its derivative translations. Apart from that, it is also the way to achieve a consistent approach to translation problems.

It was performed to detect and deal with any translation discrepancies that arise between different language versions, thus ensuring conceptual equivalence between the source and targeted language version and between all translations. This provided an additional quality control step and further ensures that data from global trials can be safely aggregated. Harmonization was performed at the same meeting as the following translation review.

Overall, the review committee was satisfied with the equivalences between the common forward translation of PSS and the English version of PSS. The common forward translation was hence regarded as the pre-final PSS-M to be tested in the next stage.

(7) Cognitive Debriefing

Cognitive debriefing involved to testing the instrument on a small group of relevant respondents or lay people in order to test alternative wording and check understandability, interpretation and cultural relevance of translation. This stage was able to highlight any items that may be inappropriate at a conceptual level and to identify any other issues that confused.

Cognitive debriefing of the pre-final PSS-M was done on a purposive sample of five respondents who were selected among caregivers who have accompanied their children for the therapy session at HUSM. Explanations regarding the purpose and procedure of cognitive debriefing were given to each respondent who agreed to participate. The respondents comprised three men and two women with ages that ranged from 25 to 40 years old. A pre-final PSS-M was given to each respondent. This section was self-administered with five minutes of allocated time.

After completing the questionnaire, the respondents were asked if they understood the statements well and if they could repeat the statements in their own words. The respondents were also asked about what came to their mind when they first read or heard a particular phrase or term and how they chose their responses. The respondents were also probed as to whether was any word that they did not understand or if there was any word or expression that they found unacceptable and offensive. Finally, when alternative words or expressions existed for one item or expression, the respondents were asked to choose the word or expression that conformed better to their usual use. In-depth interviews accomplished this information. The cognitive debriefing was completed in approximately three hours.

Additional input from the respondents including comments and suggestions were noted at the back of the respondent’s questionnaire by using a red-coloured pen. Information recorded during the pre-test session was the time duration to complete each section of the questionnaire.

(8) Review of Cognitive Debriefing Results and Finalisation

This stage was performed to incorporate the findings of the debriefing process to improve the performance of the translation. A comparison of the respondents’ interpretation of the translation with the original version to highlight and amend discrepancies will be implemented.

The result of the cognitive debriefing was explained and discussed with the review committee members. The committee evaluated all comments and suggestions given by respondents, and necessary amendments were made accordingly.

(9) Proof-Reading

The role of this stage was to consolidate PSS questionnaire and develop final version. Even though the proofreading of final translations is likely to be carried out at the end of most studies, few of the existing guidelines included it as a step in the process. The committee agreed that its inclusion was crucial because it was an important opportunity to ensure that any minor errors were corrected before the translated instrument was approved for use among the target population.

(10) Final Report

A final report was written on the process and development of the translation. This was done to explain the reasons for all the wording used throughout the translation process. This stage is essential for future translations of the same measure to be harmonized with language version previously developed.

#### Pre-Testing of Parent Satisfaction Scale Questionnaire

Pre-testing was conducted at the HUSM. A total of 30 caregivers were selected to participate in the pre-testing. This study aims to further identify any shortfalls in the translated questionnaire. The timing and overall running of the procedure were tested. We identified several shortfalls. For example, the allocated time (five minutes) was too short for the respondents. This was then increased to 10 min. From the pre-testing session, we realized that a short briefing by one of the team members before the session was helpful for the respondents to fill up the questionnaire. In the end, the final version of PSS-M was finalized and ready to be used in Phase Two of the study.

### 2.3. Statistical Analysis

Internal structure evidence of validity was provided by factor analysis and reliability [25]. In the current study, all data were entered using SPSS software version 24. The data were then transferred to R version 3.3.0 for confirmatory factor analysis (CFA) and composite reliability estimation using Raykov’s rho [26]. The caregivers’ and child’s socio-demography as well as the diagnosis process of ASD were presented as descriptive statistics.

Model fitness assessment was done using the following fit indices as recommended by Brown and Schreiber with their respective cut-off values [27,28]. The indices included the χ^2^ test (*p* > 0.05), the comparative fit index (CFI), the Tucker–Lewis fit index (TLI; ≥0.95), root mean square error of approximation (RMSEA), upper 90 percent confidence limit (≤0.08), and a standardized root mean square residual (SRMR; ≤0.08).

The model revision was considered based on factor loadings, standardized residuals (SRs), modification indices (MIs), and theoretical justification. Items with factor loadings of >0.3 were considered acceptable [23]. Parameters with SR ≥|2.58| and MI ≥3.84 were given attention for possible changes in the model specifications [29]. Multicollinearity between factors identified when correlation *r* > 0.85 [29]. For the comparison model in the current study, the Akaike information criterion (AIC) and Bayesian information criterion (BIC) were used. Those models that revealed lower values of AIC and BIC were chosen as the most fitting mode for CFA [29]. For the reliability assessment, internal reliability consistency was determined by Raykov’s rho coefficient. A reliability value of ≥0.7 was considered acceptable [23].

### 2.4. Ethical Consideration

The approval for this research was obtained from the Human Research Ethics Committee of Universiti Sains Malaysia (JEPeM Code: USM/JEPeM/17110600) and National Medical Research Register (NMMR) Malaysia (NMMR-17-2732-38655). The confidentiality of the data had been strictly maintained. Only the author and supervisors had the access to the data available. Later, the reporting and publications were carried out with no respondents’ name mentioned.

## 3. Results

### 3.1. Socio-Demographic Characteristics of the Caregivers

The mean (SD) age of caregivers was 38.39 (7.60) years. The majority of caregivers were female (79.1%), and majority had education level at diploma holder and above (77%). Table 1 shows the socio-demographic characteristics of caregivers.

### 3.2. Socio-Demographic Characteristics of the Children with ASD

The mean (SD) age of children with ASD was 7.39 (3.44) years. The majority were boys (86.4%), with co-morbidity (60%) and were registered with the Social Welfare Department (58.2%). The summary of the finding is presented in Table 2.

### 3.3. Confirmatory Factor Analysis of PSS at Specialist Centre

**Figure 2:**
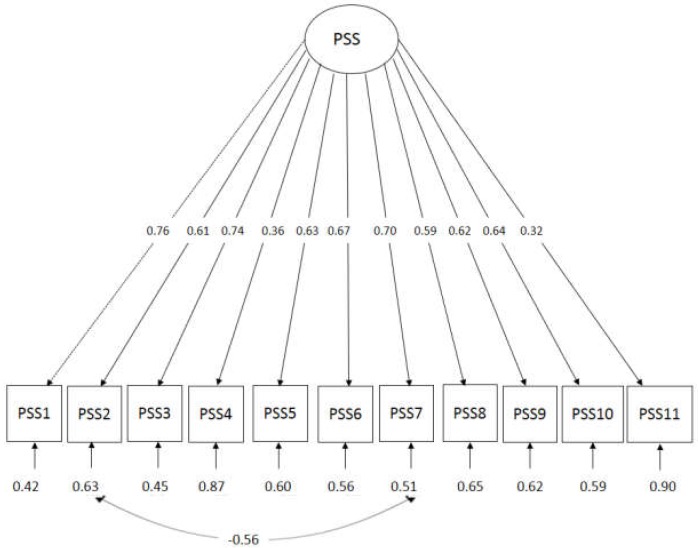
Path diagram of PSS-M confirmatory factor analysis (CFA), which indicates the interaction between items 7 and 2.

The result shows overall model fit by using identified fit indices component (Table 3). Figure 2 shows a path diagram of PSS-M CFA which indicates interactions between item 7 and 2. Thus, it was a violation of the multivariate normality assumption. Therefore, CFA was performed using a robust maximum likelihood estimator to show fitness of the model. The final model (Model 2) showed a better result where the values of AIC and BIC were lower compared to Model 1.

Table 4 shows that most of item for all factor loading were acceptable which indicated that each item contributed to the domain. The PSS-M showed good reliability, and the internal structure fit the model, with rho 0.851 and 95% Confidence Interval (CI) (0.81, 0.89).

## 4. Discussion

The ultimate aim of this study was to translate and validate the PSS to suit with the need of current target group for accessing their satisfaction on health care services. Based on the current findings, this is the first report of the validation of PSS questionnaire related to satisfaction toward healthcare services received for children with ASD among the caregivers in tertiary care, Malaysia. Overall, the questionnaire provided valid and reliable results when it was applied to caregivers of children with ASD receiving therapies in tertiary centre. The mean total score of PSS in this study was 31.99 (Table 3). The total maximum score of PSS was 44. The higher score indicates the higher level of satisfaction perceived by the caregivers on health care management of children with ASD. The fit indices of CFA model (Table 4) showed that Model 2 has a better fit based on the confirmatory fit index (CFI), Tucker–Lewis index (TLI) (≥0.95), and smaller RMSEA and SRMR. The RMSEA (*p* = 0.05) in Model 2 had a better fit as compared to original version (*p* < 0.001) [30]. Between model 1 and 2, Model 2 has better fitness acceptance, based on the lower AIC and BIC values. During model to model comparison, Model 2 was chosen because of the reduced AIC and BIC (Model 2) and the χ^2^ was significant, which indicates an improvement in model fit [29]. Both models also did not fit based on χ^2^.

Although the models did not fit the data based on robust χ^2^ like the original version [30], it is not used as a sole fit index in this study because the χ^2^ is known to be inflated by sample size and hypothesis testing as it is too stringent [19]. Thus, other fit indices were given greater weight to decide on the fitness of the models.

The overall factor loadings for the PSS-M presented in the Table 5 showed all the items were acceptable in view of recommended cut off point of reliability for factor loading >0.3 [23]. The addition correlated error item was justifiable because questions PSS 2 and PSS 7 (“I satisfied with”) were similarly worded. The composite reliability of factors based on Raykov’s rho in this newly translated questionnaire was good (construct reliability ≥0.7) (Table 4) which indicated that PSS-M has high reliability and accuracy in accessing the satisfaction level of caregivers of children with ASD [23]. The overall consistency items in the questionnaire indicate all items are measuring the same constructs. It means that if the testing process was repeated, essentially the similar results would be obtained. The results were similar to the original PSS questionnaire where it showed good factors loading among all 11-item PSS (>0.75) and corrected item-total correlations of all item >0.80. The original version was tested among parents who had children with mental health problems and had been admitted to Indiana Hospital, while the Filipino translation version of PSS which was conducted among patients receiving therapies in clinic in Manila also revealed a similar pattern of result [10,11]. The PSS-M showed excellent internal consistency reliability, well above the suggested threshold of 0.70 [23].

Apart from the psychometric properties, the rigorous methods of translation and validating processes may also positively influence the clinical utility of the PSS-M. These processes were crucial in ensuring the conceptual and linguistic equivalence retained the intended meaning of the original PSS. It is important that the concepts are well understood, language-appropriate, and measure the intended respondents in similar ways [22]. The clinicians methodically reviewed it, as did individuals skilled with languages, a public health specialist, and a statistician. It was pre-tested, and most importantly the CFA conducted showed a good model fit data with good reliability.

The ease of administration might also contribute to the clinical utility of the PSS-M. It is a self-administered questionnaire with only 11 items, and as such it is reasonably short and simple.

### Limitation of the Study

Along with the strengths, this study also had several limitations and weaknesses. The study does not include illiterate caregivers. The questionnaire was understandable for all the respondents and nobody stated any items as upsetting.

Since a large proportion of the caregivers were of the Malay ethnic group, the representation of ethnic minorities was not well presented, which limits its generalizability. The questionnaire was translated into the Malay version since Malay is the national language of Malaysia and the language most spoken by Malaysian population [31]. However, we still need to overcome the linguistic and administrative problems during the translation process which were common issues in the translation of English into the Malay version [32]. For example, the inconsistencies in the translation of certain English affixes from the original version of the questionnaire into Malay resulted in different words that might carry the same concepts. In addition, there are insufficient linguistically-trained medical experts.

The level of educational attainment of caregivers observed in this study was higher than that of the general population in Kelantan. Thus, they had the financial resources to seek early diagnosis and intervention. Families with lower levels of educational attainment may have lower average knowledge and understanding of ASD/health care management, and fewer financial resources. Lastly, the findings in this study were based on caregivers’ reports. As such, inaccurate reporting might have biased our findings. Despite these limitations, this study provides a valuable picture of vulnerable families affected by ASD in Kelantan.

## 5. Conclusions

The PSS-M model showed good construct validity and excellent absolute reliability. Thus, it can be used as a tool to determine caregiver’s satisfaction with respect to health care management for children with ASD. In addition, the validated PSS-M can also be applied to caregivers of children with other illnesses as well. It may serve as a valid indicator to measure quality of care, and may finally be used to improve the efficiency of services, primarily in local hospitals, but also for Malaysian healthcare services in general. For future studies, it is recommended to conduct cross-validation studies of PSS-M in different populations and in different health care settings.

## Figures and Tables

**Table 1:** Socio-demographic characteristics of the caregivers (*N* = 110).

Variables	*N* (%)	Mean (SD)
Age		38.39 (7.60)
Sex		
Female	87 (79.1)	
Male	23 (20.9)	
Occupation		
Professional	42 (38.2)	
Non-Professional	35 (31.8)	
Housewife/Unemployed	33 (30.0)	
Marital Status		
Single	10 (9.1)	
Married	100 (90.9)	
Main Caregiver		
Mother	60 (54.5)	
Father	8 (7.3)	
Both	42 (38.2)	
Education		
Secondary school and below	33 (30.0)	
Diploma and above	77 (70.0)	
Distance home to tertiary care		
Less than 25 km	64 (58.0)	
25 km or more	46 (41.8)	
Household income (Ringgit Malaysia; RM)		
≤RM 2000	23 (20.9)	
RM 2000 to less than RM 5000	49 (44.5)	
RM 5000 to less than RM 8000	24 (21.8)	
≥RM 8000	14 (12.7)	
Number of children		2.96 (1.71)
Availability of transportation		
Yes	103 (93.6)	
No	7 (6.4)	
Problem getting to tertiary care		
Yes	11 (10.0)	
No	99 (90.0)	
Problems in accompanying during appointments		
Yes	26 (23.6)	
No	84 (76.4)	
Medical Problems		
Yes	3 (2.7)	
No	107 (97.3)	

**Table 2:** Socio-demographic characteristics of the children with autism spectrum disorder (ASD; *N* = 110).

Variables	*N* (%)	Mean (SD)
Age		7.39 (3.44)
Gender		
Boy	95 (86.4)	
Girl	15 (13.6)	
Birth Order		1.88 (1.53)
ASD with comorbidity		
Yes	66 (60.0)	
No	44 (40.0)	
Age of caregiver start concern		2.36 (1.27)
Caregiver concerns and worries		
Speech delay		
Yes	105 (95.5)	
No	5 (4.5)	
Delay in walking		
Yes	23 (20.9)	
No	87 (79.1)	
Social problem		
Yes	63 (57.3)	
No	47 (42.7)	
Dislikes changes		
Yes	35 (31.8)	
No	75 (68.2)	
Hyperactive child		
Yes	53 (48.2)	
No	57 (51.8)	
Learning disability		
Yes	55 (50.0)	
No	55 (50.0)	
Medical problem		
Yes	8 (7.3)	
No	102 (92.7)	
Hearing Problem		
Yes	11 (10.0)	
No	99 (90.0)	
Hypersensitivity		
Yes	34 (30.9)	
No	76 (69.1)	
Sleeping problem		
Yes	32 (29.1)	
No	77 (70.0)	
No worries		
Yes	10 (9.1)	
No	100 (90.9)	
School		
Not schooling	21 (19.1)	
Government	38 (34.5)	
Non-government	51 (46.4)	
Registered with social welfare		
Yes	64 (58.2)	
No	46 (41.8)	
Age of diagnosis		5.01 (1.91)
Age when the caregiver first sought help		3.67 (2.03)

**Table 3:** Descriptive statistics of Parent Satisfaction Scale (PSS).

Statements	Mean (SD)	*n* (Percent (%))					Minimum Maximum
		0	1	2	3	4	
1. Overall, I was satisfied with the staff	3.10 (0.66)	-	6 (5.5)	1 (0.9)	79 (71.8)	24 (21.8)	1.4
2. I was satisfied with the availability of the staff	3.10 (0.68)	-	16 (14.5)	-	64 (58.2)	38 (16.7)	1.4
3. I was satisfied with the way the staff helped me understand my child’s problems	2.98 (0.93)	-	16 (14.5)	-	64 (58.2)	30 (27.3)	1.4
4. I was satisfied with the convenience of appointments with the staff	2.54 (0.92)	-	27 (24.5)	2 (1.8)	76 (69.1)	5 (4.5)	1.4
5 I was satisfied with the caring and concern the staff showed for my child	3.07 (0.89)	-	13 (11.8)	-	63 (57.3)	34 (30.9)	1.4
6. I was satisfied with how the staff treated me with respect	3.19 (0.77)	-	8 (7.3)	-	65 (59.1)	37 (33.6)	1.4
7. I was satisfied with how the staff listened to what I had to say	3.03 (0.76)	-	10 (9.1)	-	77 (70.0)	23 (20.9)	1.4
8. I was satisfied with how the staff kept me informed about changes in the care of my child.	2.88 (0.79)	-	13 (11.8)	3 (2.7)	78 (70.9)	16 (14.5)	1.4
9. I was satisfied with how the staff helped me find the services my child needed.	3.08 (0.77)	-	9 (8.2)	1 (0.9)	72 (65.5)	28 (25.5)	1.4
10. I was satisfied with how the staff included me in decision making about my child’s treatment	2.98 (0.78)	-	11 (10.0)	1 (0.9)	77 (70.0)	21 (19.1)	1.4
11. I was satisfied with the support I received from the staff.	2.04 (1.10)	-	55 (50.0)	5 (4.5)	41 (37.3)	9 (8.2)	1.4
Total PSS score	31.99 (5.74)						

**Table 4:** Fit indices of the confirmatory factor analysis (CFA) models. CFI: comparative fit index; TLI: Tucker—Lewis fit index; AIC: Akaike information criterion; BIC: Bayesian information criterion; SRMR: standardized root mean square residual; RMSEA: root mean square error of approximation.

Model	χ^2^ (df)	*P*	χ^2^_diff_ (df)	*P*	SRMR	RMSEA	90% CI	CFI	TLI	AIC	BIC
Model 1	75 (44)	<0.001			0.064	0.081	0.053, 0.108	0.90	0.87	2624	2713
Model 2	52.8 (43)	0.014	61.7 (1)	<0.001	0.056	0.050	0.000, 0.079	0.97	0.96	2598	2690

**Table 5:** Factor loading and reliability coefficient of the Malay version of the Parent Satisfaction Scale.

PSS-M Items	Factor Loading	Raykov’s rho	95% CI
PSS 1	0.76	0.851	0.81, 0.89
PSS 2	0.61		
PSS 3	0.74		
PSS 4	0.36		
PSS 5	0.63		
PSS 6	0.67		
PSS 7	0.70		
PSS 8	0.59		
PSS 9	0.62		
PSS 10	0.64		
PSS 11	0.32		

## References

[1] Oberst M.T. (1984). Patients’ perceptions of care: Measurement of quality and satisfaction. Cancer.

[2] Russell S., McCloskey C.R. (2016). Parent perceptions of care received by children with an autism spectrum disorder. J. Pediatr. Nurs..

[3] Klettlinger D., Wirfel L., Bielak B. (2015). Caregiver Perceptions of Healthcare Providers and Environments Related to Children with Autism Spectrum Disorder.

[4] Moh T.A., Magiati I. (2012). Factors associated with parental stress and satisfaction during the process of diagnosis of children with autism spectrum disorders. Res. Autism Spectr. Disord..

[5] Rivard M., Lépine A., Mercier C., Morin M. (2015). Quality Determinants of Services for Parents of Young Children with Autism Spectrum Disorders. J. Child Fam. Stud..

[6] Robert M., Leblanc L., Boyer T. (2015). When satisfaction is not directly related to the support services received: Understanding parents’ varied experiences with specialised services for children with developmental disabilities. Br. J. Learn. Disabil..

[7] APA (2013). Diagnostic and Statistical Manual of Mental Disorders.

[8] MOH (2014). Management of Autism Spectrum Disorder in Children and Adolescents.

[9] Pascoe G.C. (1983). Patient satisfaction in primary health care: A literature review and analysis. Eval. Program Plan..

[10] Gerkensmeyer J.E., Austin J.K. (2005). Development and testing of a scale measuring parent satisfaction with staff interactions. J. Behav. Health Serv. Res..

[11] Gerkensmeyer J.E. (1999). Examining Parent Satisfaction with Services for Children and Adolescents with Mental Health Problems. Ph.D. Thesis.

[12] Braiden H.-J., Bothwell J., Duffy J. (2010). Parents’ experience of the diagnostic process for autistic spectrum disorders. Child Care Pract..

[13] Chiri G., Warfield M.E. (2012). Unmet need and problems accessing core health care services for children with autism spectrum disorder. Matern. Child Health J..

[14] Karst J.S., Van Hecke A.V. (2012). Parent and family impact of autism spectrum disorders: A review and proposed model for intervention evaluation. Clin. Child Fam. Psychol. Rev..

[15] Muskat B., Greenblatt A., Nicholas D.B., Ratnapalan S., Cohen-Silver J., Newton A.S., Craig W.R., Kilmer C., Zwaigenbaum L. (2016). Parent and health care provider perspectives related to disclosure of autism spectrum disorder in pediatric emergency departments. Autism.

[16] Daniels A.M., Como A., Herguner S., Kostadinova K., Stosic J., Shih A. (2017). Autism in Southeast Europe: A Survey of Caregivers of Children with Autism Spectrum Disorders. J. Autism Dev. Disord..

[17] Siklos S., Kerns K.A. (2007). Assessing the diagnostic experiences of a small sample of parents of children with autism spectrum disorders. Res. Dev. Disabil..

[18] MOH (2015). Autism Spectrum Disorder Research in Malaysia.

[19] MOH (2017). Malaysia Health System Performance.

[20] Almeida R.S.D., Bourliataux-Lajoinie S., Martins M. (2015). Satisfaction measurement instruments for healthcare service users: A systematic review. Cad. Saude Publica.

[21] Martinez S.M., Ainsworth B.E., Elder J.P. (2008). A review of physical activity measures used among US Latinos: Guidelines for developing culturally appropriate measures. Ann. Behav. Med..

[22] Wild D., Grove A., Martin M., Eremenco S., McElroy S., Verjee-Lorenz A., Erikson P. (2005). Principles of good practice for the translation and cultural adaptation process for patient-reported outcomes (PRO) measures: Report of the ISPOR task force for translation and cultural adaptation. Value Health.

[23] Hair J.F., Black W.C., Babin B.J., Anderson R.E. (2009). Multivariate Data Analysis.

[24] Palad Y.Y., Madriaga G.O. (2014). Reliability of the Filipino version of the Parent Satisfaction Scale: A preliminary study. Hong Kong Physiother. J..

[25] Cook D.A., Beckman T.J. (2006). Current concepts in validity and reliability for psychometric instruments: Theory and application. Am. J. Med..

[26] Raykov T. (2001). Estimation of congeneric scale reliability using covariance structure analysis with nonlinear constraints. Br. J. Math. Stat. Psychol..

[27] Pituch K.A., Stevens J.P. (2015). Applied Multivariate Statistics for the Social Sciences: Analyses with SAS and IBM’s SPSS.

[28] Schreiber J.B., Nora A., Stage F.K., Barlow E.A., King J. (2006). Reporting structural equation modeling and confirmatory factor analysis results: A review. J. Educ. Res..

[29] Brown T.A. (2014). Confirmatory Factor Analysis for Applied Research.

[30] Gerkensmeyer J.E., Austin J.K., Miller T.K. (2006). Model testing: Examining parent satisfaction. Arch. Psychiatr. Nurs..

[31] Hays J. (2015). People, Population and Languages of Malaysia. http://factsanddetails.com/southeast-asia/Malaysia/sub5_4b/entry-3153.html.

[32] Quah C. (1999). Issues in the translation of English affixes into Malay. Meta J. Trad./Meta Transl. J..

